# Role of AC-cAMP-PKA Cascade in Antidepressant Action of Electroacupuncture Treatment in Rats

**DOI:** 10.1155/2012/932414

**Published:** 2012-05-28

**Authors:** Jian-hua Liu, Zhi-feng Wu, Jian Sun, Li Jiang, Shuo Jiang, Wen-bin Fu

**Affiliations:** ^1^Department of Acupuncture and Moxibustion, Guangdong Provincial Hospital of TCM, Guangzhou 510120, China; ^2^Guangdong Provincial Academy of Chinese Medical Sciences, Guangzhou 510006, China; ^3^The Second Clinical Medical College, Guangzhou University of Chinese Medicine, Guangzhou 510405, China

## Abstract

Adenylyl cyclase (AC)-cyclic adenosine monophosphate (cAMP)-cAMP-dependent protein kinase A (PKA) cascade is considered to be associated with the pathogenesis and treatment of depression. The present study was conducted to explore the role of the cAMP cascade in antidepressant action of electroacupuncture (EA) treatment for chronic mild stress (CMS)-induced depression model rats. The results showed that EA improved significantly behavior symptoms in depression and dysfunction of AC-cAMP-PKA signal transduction pathway induced by CMS, which was as effective as fluoxetine. Moreover, the antidepressant effects of EA rather than Fluoxetine were completely abolished by H89, a specific PKA inhibitor. Consequently, EA has a significant antidepressant treatment in CMS-induced depression model rats, and AC-cAMP-PKA signal transduction pathway is crucial for it.

## 1. Introduction

Depression is a common and disabling illness affecting a rising percentage of the world's population. Among the most prevalent forms of mental illness, depression is a severe, recurrent, and life-threatening illness, with about 15% of depressed patients committing suicide [1]. Although significant progress has been made in pharmacological treatment for depression over the past several decades, currently available chemical antidepressants, which exhibit 60–70% effective rate only [2], have severe side effects [3] and call for alternative treatment [4]. Acupuncture has been applied in the clinic to treat depression for a long time, and Siguan acupoints, including LI4 (*Hegu*) and LR3 (*Taichong*) acupoints, are classic acupoints in the antidepressant treatment of acupuncture. Recently, evidence from clinical and experimental studies have indicated that acupuncture may alleviate the symptoms of depression [5–7]. However, the underlying mechanism remains unknown.

 Cyclic adenosine monophosphate (cAMP) cascade, as the second messenger cascade, is considered to be associated with the pathophysiology and treatment of depression, which is a common target for several types of antidepressants. Dysfunction of the AC-cAMP-PKA cascade, including decreased G protein and cAMP level, reduced AC and PKA activity and altered PKA-mediated phosphorylation, has been observed in depressed patients [8–10]. Simultaneously, several lines of evidence clearly indicate that chronic antidepressant treatment upregulate the cAMP postreceptor signal transduction pathway at several levels. Antidepressant treatment enhanced AC/G protein coupling, expression of AC and GTP, forskolin-stimulated cAMP accumulation and levels, and activity of PKA [11–14].

In the present study, we investigate the antidepressant effect of electroacupuncture (EA) treatment in chronic mild stress (CMS)-induced depression model rats and the role of AC-cAMP-PKA postreceptor signal transduction pathway.

## 2. Methods

### 2.1. Animals

Male adult Sprague-Dawley (Experimental Animal Center, Guangdong provincial hospital of TCM, Guangzhou, China) rats, weighing 200–250 g, were used in the experiment. Upon arrival, animals were given 1 week to adjust to the new environment (20 ± 3°C, 45–60% 60–70% humidity, white noise (40 ± 10) db and 12/12 h light/dark cycle with the light from 6:00 AM to 6:00 PM), with food and water available freely prior to experimental procedures. All experimental procedures were performed during the light cycle. For all experiments, mice were randomly assigned to experimental and control groups and tested in a counterbalanced order. The animal care procedures were carried out in accordance with the National Institutes of Health Guide for the Care and Use of Laboratory Animals. Every effort was made to minimize their suffering.

### 2.2. Experimental Procedure

**Figure 1:**
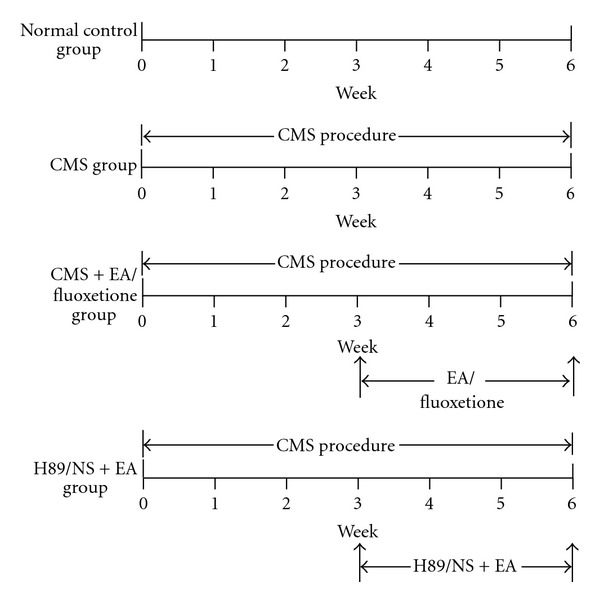
Experimental procedure.

Before stress, all rats were screened out by the openfield test in which the total scores between 70 and 120 for all experiments and then were randomly divided into the following four groups (*n* = 8 per group): (1) control group (normal control with no stress); (2) CMS group (only received stress); (3) CMS plus EA group (received stress and EA); (4) fluoxetine group (received stress and Fluoxetine). To identify the role of cAMP cascade in the antidepression effect of EA treatment, a further 24 rats (8 in each group) were divided into three groups: (5) normal saline (NS) plus EA group; (6) H89 plus EA group; (7) H89 plus Fluoxetine group. Rats in the control group did not receive any stimulation. In the CMS group, rats were exposed to chronic mild unpredictable mild stress for 6 weeks. In the EA group, rats receive CMS treatment and EA stimulation at Siguan points (bilateral *Hegu* (LI4) and *Taichong* (LR3)) once every other day for 3 weeks after CMS exposure. In the Fluoxetine group, rats receive the same treatment as the CMS group and Fluoxetine treatment everyday for 3 weeks after CMS. The groups plus H89 were conditioned with the same as the EA or Fluoxetine group except for the intracerebroventricular injection of H89 between CMS and EA/Fluoxetine delivery. The design of the experiment was shown in Figure 1.

### 2.3. CMS Procedure

The CMS protocol, as described by Willner et al. [15], consists of the sequential application of a variety of mild stresses: 24-h water deprivation, 24 h food deprivation, 30 min cage rotation, 5 min forced swim, reversal of the light/dark cycle, 5 min hot environment (40°C), a cage tilt of 45°C, white noise (100 dB), and wet bedding (100 mL of water per individual cage). The stressors were done in a random order to maximize the unpredictable nature of the stressors. The CMS procedure was carried out in stressed animals once per day for 3 weeks.

### 2.4. Open-Field Test

The open-field test was performed as previously described [15] and was carried out before stress (day 0), 3 weeks after stress (day 22), and 4, 5, and 6 weeks after stress. In the openfield test, rats were placed at the center rectangular arena side walls, which was a four-sided 100 × 100 × 40 cm^3^ wooden box with the walls painted black. The floor of the box was divided into 16 squares. The room was in a dimly lit with a video camera above the center of the floor. Each animal was placed in the center of the box and was allowed to explore freely for 3 min. During the test time the number of crossings (defined as at least three paws in a quadrant) and rearings (defined as the animal standing upright on its hind legs) was measured. After the test of each animal, the test box was cleaned with a 10% ethanol solution and water to remove any olfactory cues.

### 2.5. Sucrose Intake Test

The sucrose intake test was performed on days 0, 22, and 43. Prior to the start of the test, animals were trained to consume 1% sucrose solution. They were habituated for 48 hours to two bottles: one with 1% sucrose (Sigma), the other with tap water, followed by a period of 24 h without any food and water available, and a 1-h exposure to the two identical bottles again for testing fluid consumption. In order to have concordance measure for all groups, each rat in control group was randomly selected out and kept housed individually at the beginning of this test. Two-bottle tests for each cage were adopted throughout the procedure. Sucrose solution consumption was recorded by calculating volume of test solution.

### 2.6. Measurement of Body Weight

Body weight in all rats was measured every week throughout the experiment.

### 2.7. EA and Drug Treatment

For EA stimulation, the rat was slightly immobilized in a small cylindrical container so that the movement of the rat's head restrained while the body could move freely. EA at Siguan acupoints, bilateral Hegu (LI4), and Taichong (LR3) were performed for 30 min once every other day. Location of the acupoints was determined by comparative anatomy. LI4 is located on the dorsum of the forelimb, between the 1st and 2nd metacarpal bones, approximately in the middle of the 2nd metacarpal bone on the radial side and LR3 is located on the dorsum of the claw of the hindlimb, proximal to the first metatarsal space. Stainless-steel needles of 0.18 mm diameter and 15 mm length were inserted into the acupoints. The electrical stimulation was from a medical EA apparatus (model G6805-2, Shanghai, China). The stimulation parameters were of frequency 2 and 20 Hz, alternatively, strong enough to only elicit slight twitches of the limbs. In order to exclude the possibility of stress induced by animal fixation, all groups were slightly immobilized in the same container for 30 min. For the Fluoxetine treatment, animals were given Fluoxetine (Eli Lilly, USA) at a dose of 1.8 mg/kg i.g. (dissolved in sterile 0.9% physiological saline) daily prior to the immobilized period.

### 2.8. Intracerebroventricular Injection

Rats were anesthetized with 10% hloralhydrate (350 mg/kg, i.p.) and placed in a rat brain stereotaxic apparatus (Narishige, Japan). After a midline scalp incision, the head position was adjusted to place bregma and lambda in the same horizontal plane. A small hole (0.8 mm posterior to bregma, 1.5 mm lateral to midline) was drilled on the skull. A stainless-steel guide cannula (22 gauge, ID 0.58 mm, and OD 0.90 mm) was placed unilaterally in the lateral cerebral ventricle at a depth of 4.0 mm and fixed with dental cement onto the skull, serving as a guide for the accurate insertion of a Internals cannula (extending 0.5 mm below the tip of the guide cannula). To prevent clogging or infection of the brain tissue, a dummy cannula (OD 0.56 mm) was always placed in the guide cannula as a cap for covering the guide cannula except the duration of injection. The rats were given about 3 days to recover completely from the surgery. The animals with implanted cannula were placed in transparent plastic cages and were freely moving throughout the perfusion. The Osmotic mini pump filled with either H89 or normal saline (NS) was connected through a PE tube (150 mm length) to the internal cannula. H89 (dissolved in sterile saline, 10 *μ*M, 5 *μ*L) or NS (0.9%, 5 *μ*L) was microinjected into the lateral cerebral ventricle through the cannula at a flow rate of 1uL/min. EA or Fluoxetine was applied at least 30 min after the perfusion procedures.

### 2.9. Assay of AC Activity

The dissected hippocampus was homogenized (1 : 40 (w/v)) in ice-cold buffer (320 mM sucrose, 1.6 mM EGTA, and 2 mM Tris pH 7.4) using 10 strokes with a Teflon tissue grinder and centrifuged (1000 rpm, 10 min, 4°C), and the supernatant was centrifuged again (20, 000 rpm, 20 min, 4°C). The pellet was resuspended in ice-cold assay buffer. The AC assay was performed after 10 min preincubation at 30°C in a reaction mixture (final volume, 500 *μ*L) containing 80 mM Tris pH 7.4, 2 mM EGTA, 3 mM MgCl2, 0.5 mM IBMX, and 5 *μ*M forskolin. The reaction was initiated by addition of ATP to a final concentration of 200 *μ*M and then incubated (10 min at 30°C) and stopped by boiling for 3 min. The samples were centrifuged (3000 rpm, 10 min), and cAMP accumulation was quantified in 50 *μ*L supernatant aliquots by using the [^3^H] cAMP assay kit (China Institute of Atomic Energy, China).

### 2.10. Assay of cAMP Level

Hippocampus was homogenized in 1 : 40 (w/v) in ice-cold buffer and centrifuged (1000 rpm, 10 min, and 4°C) as described above. The supernatants were incubated by boiling for 5 min and then centrifuged (15,000 rpm, 10 min). cAMP accumulation was quantified in 50 uL supernatant aliquots by using a [^3^H]cAMP assay kit (China institute of atomic energy, China).

### 2.11. Assay of PKA Activity

PKA activity was assayed using radioactive PKA assay kit (Promega, USA) following the manufacturer's instructions. Protein/sample (50 mg) was used for kinase activity.

### 2.12. Statistical Analysis

Data were presented as mean ± standard error of the mean (SEM). Differences between groups were considered to be statistically significant for *P* < 0.05. The significance of differences was determined using the one-way ANOVA followed by least significant difference (LSD) as post hoc multiple comparisons test. When two factors were assessed, the significance of differences was determined using two-way ANOVAs.

## 3. Results

### 3.1. Openfield Test

**Figure 2:**
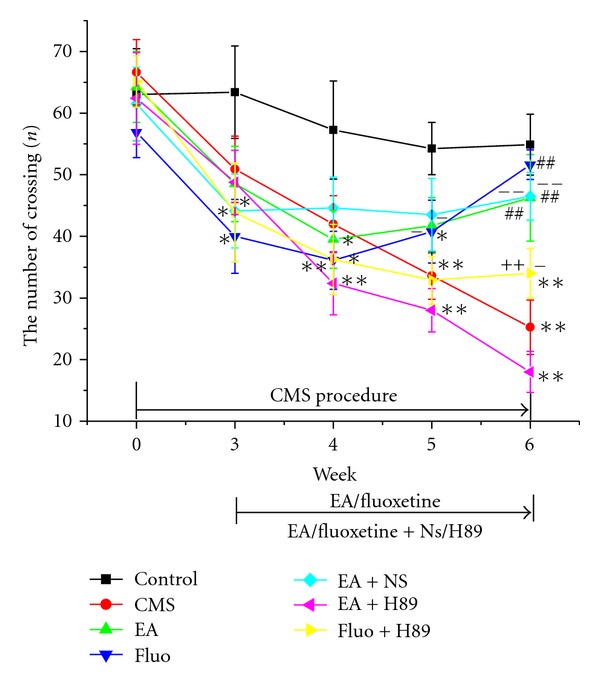
Effect of EA or Fluoxetine on the number of crossing in openfield test. *P < 0.05 and **P < 0.01 versus control group, respectively; ^##^ *P* < 0.01 versus CMS group;  ^++^ *P* < 0.01 versus Fluoxetine group; ^−^ *P* < 0.05 and ^−−^ *P* < 0.01 versus EA + H89 group, respectively.

**Figure 3:**
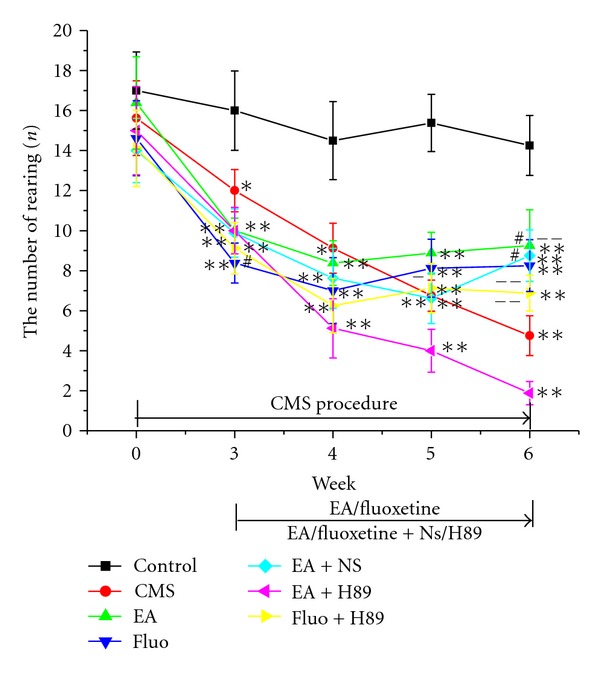
Effect of EA or Fluoxetine on the number of rearing in openfield test. *P < 0.05 and **P < 0.01 versus control group, respectively, ^#^ *P* < 0.05 versus CMS group, ^−^ *P* < 0.05 and  ^−−^ *P* < 0.01 versus EA + H89 group, respectively.

All stress groups began to show behavior deficit in the 3rd week, indicating obvious difference with the control group. However, there was no remarkable difference among all stress groups. In the sixth week, EA (46.25 ± 7.03, *P* < 0.01) and Fluoxetine (51.62 ± 2.41, *P* < 0.01) as well as EA + NS (46.5 ± 3.86, *P* < 0.01) treatment leaded to increase in the number of crossing, indicating significant difference with CMS group (25.25 ± 4.42). Enhanced effects induced by EA or Fluoxetine were reversed by H89 pretreatment, respectively (18.0 ± 3.32 versus 46.25 ± 7.03, *P* < 0.01; 34.0 ± 4.03 versus 51.62 ± 2.41, *P* < 0.01, resp.) (Figure 2). Changes in the number of rearing were similar to the crossing (Figure 3).

### 3.2. Sucrose Intake

**Figure 4:**
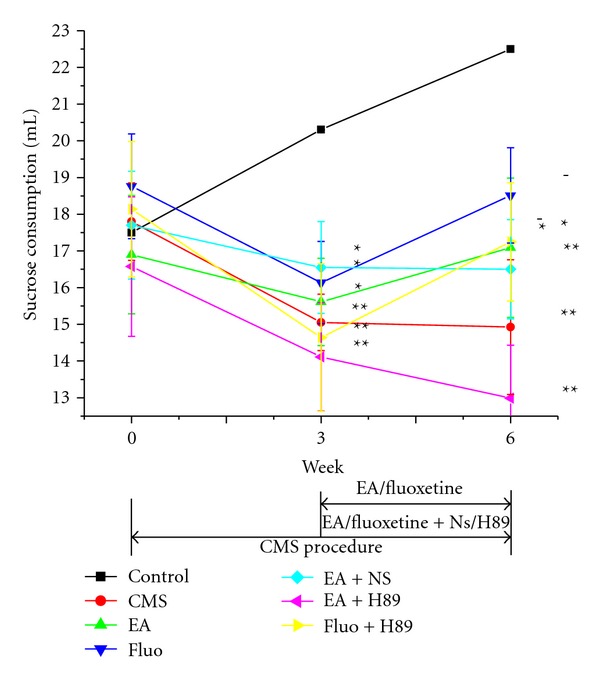
Effect of EA or Fluoxetine on sucrose intake.  *P < 0.05, and **P < 0.01 versus control group, respectively;  ^−^ *P* < 0.05 versus EA + H89 group.

Sucrose intake in all stress groups decreased significantly in the 3rd week and was much less than the control group (*P* < 0.05 or *P* < 0.01). However, there was no remarkable difference among all stress groups. EA (18.09 ± 1.90) or Fluoxetine (18.51 ± 1.30) treatment leaded to increase in sucrose intake in the sixth week, however, not indicating significant difference with CMS group (14.93 ± 1.83). H89 pretreatment inhibited obviously increase in sucrose intake induced by EA (12.99 ± 1.45 versus 18.09 ± 1.90, *P* < 0.05), but had no effect on the Fluoxetine (17.25 ± 1.61 versus 18.51 ± 1.30, *P* > 0.05) (Figure 4).

### 3.3. Body Weight

**Figure 5:**
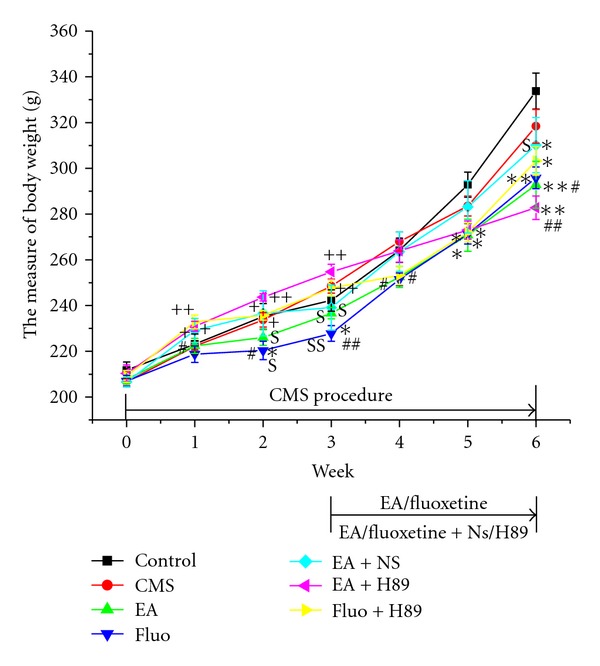
Effect of EA or Fluoxetine on body weight. *P < 0.05, **P < 0.01 versus control group, respectively; ^#^ *P* < 0.05, and ^##^ *P* < 0.01 versus CMS group, respectively; ^+^ *P* < 0.05 and ^++^ *P* < 0.01 versus Fluoxetine group, respectively.

Body weight in all groups increased during the whole experiment. EA (292.75 ± 10.23 versus 318.38 ± 7.38, *P* < 0.05), and Fluoxetine (295.88 ± 4.74 versus 318.38 ± 7.38, *P* < 0.05) group had less body weight than CMS group at the end of the last week. H89 + EA or H89 + Fluoxetine group had similar body weight gain to EA or Fluoxetine group (Figure 5).

### 3.4. AC-cAMP-PKA Cascade

**Figure 6:**
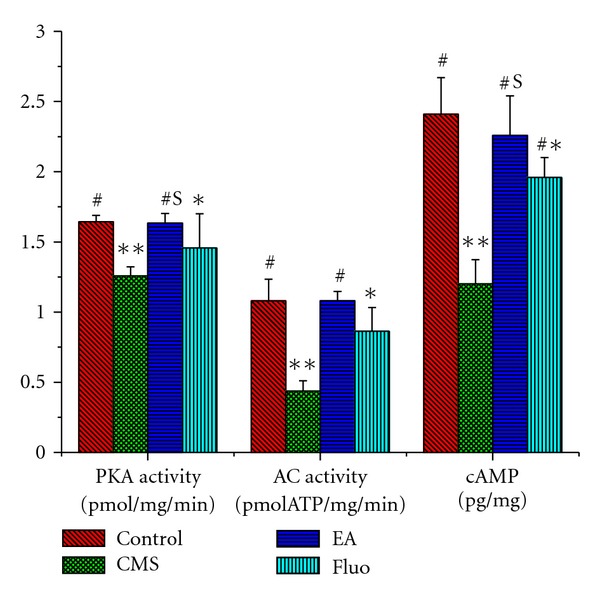
Effect of EA or Fluoxetine on AC transformation ratio, cAMP level, and PKA activity. *P < 0.05 versus control group; ^##^ *P* < 0.01 versus CMS group; ^S^ *P* < 0.05 versus Fluoxetine group.

CMS produced a significant decrease in the ratio of AC transformation compared with the control group (0.44 ± 0.07 versus 1.08 ± 0.15, *P* < 0.01), which was reversed by EA or Fluoxetine treatment (1.08 ± 0.07, 0.86 ± 0.17, *P* < 0.01, *P* < 0.01). Changes in cAMP level and PKA activity were similar to AC (Figure 6).

## 4. Discussion

Because of good predictive validity, face validity and construct validity, the CMS model has become the most extensively used animal model of depression [16]. In this study, the results showed that CMS induced obvious behavior deficits and decrease in sucrose intake, which were reversed by EA or fluoxetine, suggesting that EA may be as effective as antidepressants in treating depression. Simultaneously, we observed that changes in body weight were different from behavior and sucrose intake. EA and Fluoxetine treatment had less body weight than CMS and control group, which was not reversed by a specific PKA inhibitor H89, suggesting that EA or Fluoxetine had no effect on body weight in CMS-induced depression model rats. Body weight has been viewed as a marker in depression study, and CMS causes about 0–10% loss of body weight [16]. However, some researches show that CMS rats have body weight gain and antidepressants, including Fluoxetine and clomipramine, have even less body weight than CMS and normal control group [7, 17]. A recent study also shows that EA treatment or EA combined with clomipramine has similar body weight gain to the CMS and control group [7]. Michelson et al. observed changes in weight during a 1-year trial of Fluoxetine and found that acute therapy during initial 4 weeks with Fluoxetine is associated with modest weight loss and fluoxetine or placebo produced weight gain after 50-week therapy [18]. Therefore, whether body weight measurement is an important marker in depression study needs more sufficient evidence.

In the present study, the results showed that CMS induced downregulation of AC-cAMP-PKA cascade, which was reversed by EA and Fluoxetine treatment. AC-cAMP-PKA cascade, as the second messenger cascade, has been implicated in the pathophysiology of depression and antidepressant action. Receptor activation induced by ligand (hormones, neurotransmitters and growth factors, etc.) contribute to the generation of cAMP via the stimulation of AC by the G-protein subtype Gs, which then leads to the activation of PKA. PKA is responsible for regulatory effects on cellular functions through the phosphorylation of specific target proteins. Amongst the substrates of PKA is the cAMP response element binding protein (CREB), a transcription factor that mediates the actions of cAMP cascade on gene expression and exhibits an increase in its ability to modulate transcriptional activity, in the dephosphorylated form. Modulation of this transcription factor and its target genes results in the cellular adaptations underlying the antidepressant actions [19–21]. Dysfunction of the AC-cAMP-PKA cascade, including decreased G protein and cAMP level, reduced AC and PKA activity and altered PKA-mediated phosphorylation, have been observed in depressed patients [8–10]. Simultaneously, evidence clearly indicates that chronic antidepressant treatment upregulates the cAMP postreceptor signal transduction pathway at several levels. Antidepressant treatment enhanced AC/G protein coupling, which contributes to increased AC activity, and expression of AC and GTP and forskolin-stimulated cAMP accumulation [11–14]. An important evidence about the role of cAMP cascade in antidepressant action comes from rolipram, a phosphodiesterase inhibitor, which inhibits the cAMP metabolism. Rolipram has been reported to have antidepressant effects in clinical trials and is not in clinical use because of its side effects [22]. Moreover, Levels and activity of PKA are enhanced by antidepressant treatment [14]. An increase of PKA levels is observed in the crude nuclear fraction following antidepressant administration, indicating a translocation of PKA into the nucleus [12]. The nuclear translocation of PKA suggests that antidepressant treatments may recruit the cAMP cascade to regulate its target gene expression, such as brain-derived neurotrophic factor (BDNF). These results are in agreement with the present study.

Furthermore, an interesting result was observed in this study. Pretreatment of H89, a specific PKA inhibitor, abolished completely the antidepressant effect of EA, and the depressive-like behavior and sucrose intake as well as body weight in EA + H89 group were all much less than CMS group, suggesting that PKA activity is crucial for antidepressant effect of EA treatment. Furthermore, the dosage of H89 administration in this study may be sufficient to inhibit completely the PKA activity in the hippocampus. However, PKA activity in CMS may partly decrease. So H89 + EA had even more depressed sign than CMS.

At the same time, H89 did not influence the antidepressant action of Fluoxetine, suggesting other signal transduction pathway may be involved in it. Tronson et al. find that intrahippocampal injection of PKA inhibitor Rp-cAMPS has no remarkable effect on depression-like behavior in mice [23]. Chronic Fluoxetine treatment exerts a more marked effect on phospho-CREB (pCREB) in hippocampus and prefrontal/frontal cortex. However, desipramine and reboxetine, but not Fluoxetine, increase consistently the activity of nuclear PKA, suggesting that PKA does not seem to account for increase of pCREB induced by Fluoxetine [24]. Moreover, various kinds of studies have demonstrated that, in addition to cAMP-PKA cascade, calcium/calmodulin (CaM)-dependent kinases (CaMK) and mitogen-activated protein kinases (MAPK) cascades are involved in the selective serotonin reuptake inhibitors (SSRIs)-induced antidepressant actions [24, 25]. Consequently, although Fluoxetine may upregulate the AC-cAMP-PKA cascade, dysfunction of PKA did not abolish the antidepressant actions.

In conclusion, EA has a significant antidepressant treatment in CMS-induced depression model rats, as effective as Fluoxetine, and AC-cAMP-PKA postreceptor signal transduction pathway may be crucial for it.

## References

[1] Song L, Che W, Wei WM (2006). Impairment of the spatial learning and memory induced by learned helplessness and chronic mild stress. *Pharmacol Biochem Behav*.

[2] Dording CM, Mischoulon D, Petersen TJ (2002). The pharmacologic management of SSRI-induced side effects: a survey of psychiatrists. *Annals of Clinical Psychiatry*.

[3] Rosen RC, Marin H (2003). Prevalence of antidepressant-associated erectile dysfunction. *Journal of Clinical Psychiatry*.

[4] Pohl A, Nordin C (2002). Clinical and biochemical observations during treatment of depression with electroacupuncture: a pilot study. *Human Psychopharmacology*.

[5] Fu WB, Fan L, Zhu XP (2006). Clinical research on acupuncture treatment of depressive neurosis by using liver-function-regulating method. *Zhen Ci Yan Jiu*.

[6] Fu WB, Fan L, Zhu XP (2009). Depressive neurosis treated by acupuncture for regulating the liver—a report of 176 cases. *Journal of Traditional Chinese Medicine*.

[7] Yu J, Liu Q, Wang YQ (2007). Electroacupuncture combined with clomipramine enhances antidepressant effect in rodents. *Neuroscience Letters*.

[8] Dowlatshahi D, MacQueen GM, Wang JF, Reiach JS, Young LT (1999). G protein-coupled cyclic AMP signaling in postmortem brain of subjects with mood disorders: effects of diagnosis, suicide, and treatment at the time of death. *Journal of Neurochemistry*.

[9] Shelton RC, Manier DH, Sulser F (1996). cAMP-dependent protein kinase activity in major depression. *American Journal of Psychiatry*.

[10] Cowburn RF, Marcusson JO, Eriksson A, Wiehager B, O’Neill C (1994). Adenylyl cyclase activity and G-protein subunit levels in postmortem frontal cortex of suicide victims. *Brain Research*.

[11] Ozawa H, Rasenick MM (1991). Chronic electroconvulsive treatment augments coupling of the GTP-binding protein G(s) to the catalytic moiety of adenylyl cyclase in a manner similar to that seen with chronic antidepressant drugs. *Journal of Neurochemistry*.

[12] Nestler EJ, Terwilliger RZ, Duman RS (1989). Chronic antidepressant administration alters the subcellular distribution of cyclic AMP-dependent protein kinase in rat frontal cortex. *Journal of Neurochemistry*.

[13] Jensen JB, Mikkelsen JD, Mørk A (2000). Increased adenylyl cyclase type 1 mRNA, but not adenylyl cyclase type 2 in the rat hippocampus following antidepressant treatment. *European Neuropsychopharmacology*.

[14] Perez J, Tinelli D, Brunello N, Racagni G (1989). cAMP-dependent phosphorylation of soluble and crude microtubule fractions of rat cerebral cortex after prolonged desmethylimipramine treatment. *European Journal of Pharmacology*.

[15] Willner P, Towell A, Sampson D, Sophokleous S, Muscat R (1987). Reduction of sucrose preference by chronic unpredictable mild stress, and its restoration by a tricyclic antidepressant. *Psychopharmacology*.

[16] Willner P (1997). Validity, reliability and utility of the chronic mild stress model of depression: a 10-year review and evaluation. *Psychopharmacology*.

[17] Wu X, Alberico SL, Moges H (2012). Pulsed light irradiation improves behavioral outcome in a rat model of chronic mild stress. *Lasers in Surgery and Medicine*.

[18] Michelson D, Amsterdam JD, Quitkin FM (1999). Changes in weight during a 1-year trial of fluoxetine. *American Journal of Psychiatry*.

[19] Duman RS, Malberg J, Nakagawa S, D’Sa C (2000). Neuronal plasticity and survival in mood disorders. *Biological Psychiatry*.

[20] Shaywitz AJ, Greenberg ME (1999). CREB: a stimulus-induced transcription factor activated by a diverse array of extracellular signals. *Annual Review of Biochemistry*.

[21] Silva AJ, Kogan JH, Frankland PW, Kida S (1998). CREB and memory. *Annual Review of Neuroscience*.

[22] O’Donnell JM (1993). Antidepressant-like effects of rolipram and other inhibitors of cyclic adenosine monophosphate phosphodiesterase on behavior maintained by differential reinforcement of low response rate. *Journal of Pharmacology and Experimental Therapeutics*.

[23] Tronson NC, Schrick C, Fischer A (2008). Regulatory mechanisms of fear extinction and depression-like behavior. *Neuropsychopharmacology*.

[24] Tiraboschi E, Tardito D, Kasahara J (2004). Selective phosphorylation of nuclear CREB by fluoxetine is linked to activation of CaM kinase IV and MAP kinase cascades. *Neuropsychopharmacology*.

[25] Duman CH, Schlesinger L, Kodama M, Russell DS, Duman RS (2007). A role for MAP kinase signaling in behavioral models of depression and antidepressant treatment. *Biological Psychiatry*.

